# Spermatorrœa

**Published:** 1879-06

**Authors:** 


Spermatorrhœa: Its Causes, Symptoms, Results and Treatment. By Roberts Bartholow, A.M., M.D., Professor of the Theory and Practice of Medicine, and of Clinical Medicine, in the Medical College of Ohio, etc. Wm. Wood & Co., New York. 1879.

In the publication of this work, the first step has been taken toward a salutary and rational understanding of this often deplorable disease. Arising, as the trouble does, from causes that render the victim particularly reserved and shy in employing professional assistance, it has presented a wide field for the disgraceful operations of advertising quacks. Could the work be brought prominently to the notice both of the profession and the laity, much benefit could be given and disaster prevented. The author's style is plain and comprehensive, and the subject is treated in a systematic and scientific manner.

In the consideration of the subject the author has made the following division: 1. True Spermatorrhœa. Its causes—physical, moral. Its symptoms; its results. 2. Spermatorrhœa as a symptom. 3. False or imaginary Spermatorrhœa. 4. Treatment.

Masturbation is considered the chief cause, though at times it may be secondary, as various inducements to venereal excitement may arise from prolonged sitting, as at school, and other predisposing influences to pelvic congestions and genito-urinary irritation.

Altogether the book is well worthy a careful perusal, and should be read and distributed generally. No library—no family should he without it.

